# Protocol for simulating the effect of THz electromagnetic field on ion channels

**DOI:** 10.1002/qub2.94

**Published:** 2025-03-21

**Authors:** Lingfeng Xue, Zigang Song, Qi Ouyang, Chen Song

**Affiliations:** ^1^ Center for Quantitative Biology Academy for Advanced Interdisciplinary Studies Peking University Beijing China; ^2^ School of Life Sciences Peking University Beijing China; ^3^ School of Physics Peking University Beijing China; ^4^ Peking‐Tsinghua Center for Life Sciences Academy for Advanced Interdisciplinary Studies Peking University Beijing China

**Keywords:** calcium channel, frequency spectrum, ion channel, terahertz

## Abstract

Terahertz (THz) electromagnetic fields are increasingly recognized for their crucial roles in various aspects of medical research and treatment. Recent computational studies have demonstrated that THz waves can modulate ion channel function by interacting with either the channel proteins or the bound ions through distinct mechanisms. Here, we outline a universal simulation protocol to identify the THz frequencies that may affect ion channels, which consists of frequency spectrum analysis and ion conductance analysis. Following this protocol, we studied the effect of the THz field on a Ca_V_ channel and found a broad frequency band in the 1–20 THz range. We believe that this protocol, along with the identified characteristic frequencies, will provide a theoretical foundation for future terahertz experimental studies.

The terahertz (THz, 10^12^ Hz) electromagnetic field is an emerging technology with significant potential in medical diagnostics and treatment [1]. As an imaging modality, THz waves have been employed to detect various types of cancers, including breast tumors and skin cancer [2, 3]. Additionally, THz technology has been explored for the diagnosis and treatment of a range of diseases such as thyroid nodules and Alzheimer’s disease [4].

The biological effects of THz fields can be broadly categorized into thermal and nonthermal effects. The thermal effect arises from the absorption of THz radiation by biological tissues, resulting in the conversion of electromagnetic energy into heat. In contrast, the nonthermal effects encompass a range of biological responses. For instance, in vitro studies have shown that THz radiation can induce morphological abnormalities in neuron cell membranes and alter intracellular structures [5]. Similarly, studies on blood cells revealed that THz radiation can affect the permeability of erythrocyte membranes [6]. Additionally, when THz radiation was applied to mammalian stem cells, certain genes were activated while others were repressed, suggesting its potential role in cellular reprogramming [7]. Moreover, THz radiation was found to induce DNA double strand breaks, leading to DNA damage in skin tissues [8]. Mathematical modeling studies have also indicated that THz radiation may induce resonance effects in DNA, leading to modifications in gene expression [9]. These effects are thought to be mediated by the fact that many biomolecules exhibit inter‐ and intramolecular motions within the THz frequency range, contributing to both the thermal and nonthermal interactions of THz fields.

Computational methods have been applied to illustrate the mechanisms of THz effect on biological molecules. For example, molecular dynamics (MD) simulations were performed to explore the mechanism of membrane electroporation under the THz field [10]. Also, analysis of MD simulation results revealed the vibrational landscape of proteins in the THz frequency region [11]. For DNA, a study using MD simulations demonstrated that a terahertz stimulus at 44.0 THz can accelerate the unwinding process of DNA duplexes [12]. Similarly, steered MD simulations showed that THz waves can promote the unfolding of the double helix of the RNA hairpin [13].

Recently, several groups have investigated the effects of THz fields on ion channels. For example, it was found that a 5.6 μm (53.5 THz) field can increase potassium current [14] (Figure 1A). MD simulations on the KcsA channel revealed that a THz field with a frequency of 51.87 THz significantly increased potassium current, which corresponds to the vibration of –C=O groups in the selectivity filter (SF) region [15]. Similarly, MD simulations on the Ca_V_Ab channel showed that a frequency of 42.55 THz enhanced the channel’s permeability, corresponding to the stretching mode of the carboxylate group at the SF [16]. These findings indicate that THz fields can modulate ion channels by affecting specific functional groups of proteins (Figure 1B).

**FIGURE 1:**
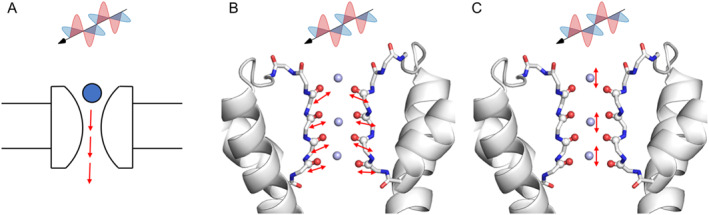
Effect of THz fields on ion channels through different mechanisms. (A) THz fields can affect the ion conductance. (B) THz fields affect the vibration motions of carbonyl bonds at the SF. (C) THz fields affect the vibration motions of ions at the SF. SF, selectivity filter.

Our recent studies further demonstrated that the ion oscillation frequencies also lie within the THz range, and the application of specific THz frequencies can regulate ion conductance [17]. Based on these findings, we proposed a new mechanism for the regulation of ion channels (Figure 1C), wherein the applied THz field directly influences ion motion, accelerating the ions as they permeate the channel. It was unclear whether the enhanced ion conductance was due to a thermal effect. To investigate this, we applied electric fields in different directions. We found that ion conductance increased only when the THz field was applied in the *z*‐direction (perpendicular to the membrane), whereas fields applied in the *x*‐ or *y*‐directions had no significant effect. This direction dependence indicates that the observed enhancement of ion conductance by THz fields is not simply a thermal effect, thereby further sparking interest in the study of THz interactions with ion channels.

To advance the study of THz effects on ion channels, here, we summarize a universal simulation protocol designed to identify THz frequencies that may influence ion conductance (Figure 2). This protocol includes the following steps:

1. Select the ion channel of interest, construct the MD simulation system, and perform adequate equilibrium simulations. CHARMM‐GUI is a convenient tool for this step [18]. An open‐state channel is preferred, as we may want to validate the effect of the THz field by simulating the conductance of the channel in the fourth step.
2. Initiate MD simulations without any applied electric field. During these simulations, the velocities of all the atoms, or the atoms of interest, are sampled at an interval of $\frac{1}{2f_{\max}}$, where $f_{\max}$ is the highest frequency in the intended spectrum. In our case, the highest frequency is 100 THz, so the velocities are recorded every 5 fs, allowing the high‐frequency motions to be fully captured.
3. Perform frequency spectrum analysis on the velocities of key ions and protein functional groups to identify their characteristic frequencies. The key ions refer to ions that are bound within the channel, while the key functional groups of proteins refer to the atoms that directly interact with the permeating ions. The frequency spectrum analysis can be accomplished using the FFT (fast Fourier transform) analysis. For instance, one can use the numpy.fft function in Python to execute the analysis. The FFT can identify the characteristic frequencies by transforming the velocity time series data into the frequency domain, where peaks in the frequency spectrum represent the dominant vibrational modes of the ions or atoms. It is crucial to pay special attention to the ion‐binding sites along the permeation pathway. This includes not only the chemical groups of the ion channels that form these binding sites but also the bound ions themselves, as these often exhibit motions that fall within the THz frequency range, making them likely targets for modulation by THz fields.
4. Using these characteristic frequencies, we applied THz fields to assess their impact on ion conductance. This step can be further divided into two parts:
  - Simulate the ion conduction in the absence of the THz field. We apply a constant electric field in the *z*‐direction (membrane normal) to calculate the conductance of the channel, using the formula $g=\frac{I}{U}=\frac{Nq}{tEL_{z}}$, where g is conductance, I is current, which is calculated by the number of ion permeation events N for a given period t, and U is transmembrane voltage, which is proportional to the electric field E and the *z*‐direction box size $L_{z}$. The calculated conductance should be compared to the experimental values to validate the simulation parameters and protocol.
  - Simulate ion conduction in the presence of the THz field by applying an additional THz electric field, in addition to the constant electric field in the *z*‐direction. The magnetic field can be neglected due to its minimal impact compared to the electric field on ion channels. Apply the THz field in the *x*‐, *y*‐, and *z*‐directions, respectively. The current GROMACS package does not support an additional THz field along the same direction as the constant electric field, probably because this scenario was not considered during the algorithm design. Therefore, we developed a plug‐in to introduce an extra constant electric field by modifying the source code of GROMACS to include additional constant electric field parameter settings (Please refer to the GitHub repository, ComputBiophys/GMX_ElectricField_Plugin for more details). Using this plug‐in, one can perform simulations with both THz and constant electric fields along the same direction to determine the ion conductance of the channel.
5. Compare the simulated ion conductances from the simulations with and without the THz field to assess whether the selected THz frequency can significantly alter ion conductance. It is important to note that both the frequency and the direction of the THz field are crucial factors, likely corresponding to the motion characteristics of the chemical groups or ions analyzed in the third step above.

**FIGURE 2:**
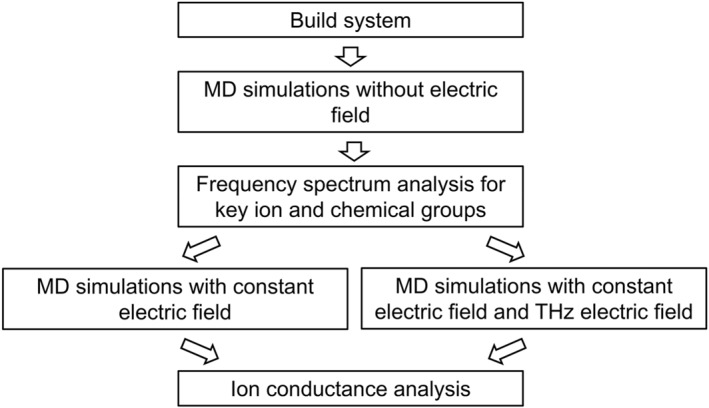
The overall simulation and analysis protocol for studying the effect of THz fields on ion channels.

Overall, this computational protocol allows us to identify the characteristic frequencies of key ions and functional groups, as well as to evaluate the effects of THz fields on channel conductance at these frequencies. Previous studies mainly focused on the key functional groups in proteins [15, 16], while our protocol also emphasizes the motion of ions in the channel. Moreover, our protocol offers a novel approach to assess the effect of the THz electric field using a newly developed GROMACS plug‐in.

Using the above protocol, we calculated the THz frequencies for the Ca_V_1.3 channel (Figure 3). We utilized a closed‐state Ca_V_1.3 structure [19] for the simulations, as an open‐state structure is not currently available. To assess model dependency, we adopted two different calcium models: the default CHARMM model and our recently developed multi‐site calcium model [20]. Following the protocol steps 1–3, we identified the characteristic frequencies for Ca^2+^ ions at the SF to be in the range of 1–20 THz for both models (Figure 3A,C). With the CHARMM calcium model, the peak frequency was approximately 8 THz, while simulations using the multi‐site calcium model revealed peak frequencies at 2, 5, and 16 THz. Although the results from the two models were not identical, both indicate that THz frequencies in the range of 1–20 THz may influence Ca^2+^ conductance. Note that the artificial peak at 69 THz, observed with the multi‐site calcium model, was attributed to bond vibrations of pseudo atoms and should be disregarded. For the carboxylate groups in the SF of Ca_V_1.3, we identified two characteristic frequencies at 42.6 and 48.5 THz (Figure 3B and D), which are very close to previous results observed in Na_V_ channels. Ion permeation simulations (steps 4–5) were not conducted due to the unavailability of an open‐state structure for Ca_V_.

**FIGURE 3:**
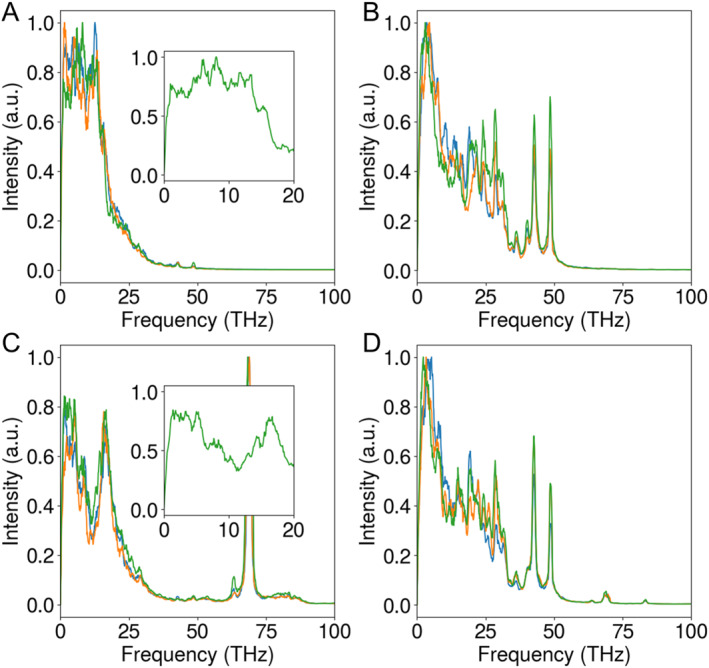
Frequency spectrum of Ca^2+^ ions (A) and carboxylate oxygen (B) at the SF of Ca_V_1.3 with the default CHARMM calcium model. (C, D) Similar to A–B, but obtained with the multi‐site calcium model. The blue, orange, and green lines represent the frequency spectra for velocities in the *x*‐, *y*‐, and *z*‐directions, respectively. SF, selectivity filter.

In summary, we present a table (Table 1) detailing the likely characteristic THz frequencies and the corresponding chemical groups of voltage‐gated ion channels that may be affected. For K^+^ ions in K_V_1.2, the characteristic frequencies are 1.4, 2.2, and 2.9 THz. For Na^+^ ions in Na_V_1.5, the characteristic frequency range is broad, spanning 1–10 THz, with notable peaks at 2.5 and 10 THz. Similarly, for Ca^2+^ ions in Ca_V_1.3, the characteristic frequency ranges from 1 to 20 THz, with peaks at 2, 5, 8, and 16 THz. In addition, the chemical groups that bind the permeating ions in the SF of ion channels show important frequencies too, including the carbonyl bond stretching in K_V_1.2 with a frequency of 51 THz and the carboxylate bond stretching in sodium and calcium channels with frequencies of 42.5 and 48.6 THz, respectively. Note that the key groups responsible for ion binding are different in the three types of channels. In K_V_ channels, the backbone carboxyl oxygen atoms directly coordinate with K^+^. In contrast, in Na_V_ and Ca_V_ channels, the aspartate and glutamate residues coordinate with Na^+^ or Ca^2+^. These differences lead to the different characteristic frequencies of ions in the channels.

**TABLE 1:** Characteristic frequencies of ions and chemical groups at the SF of ion channels.

Channel	Characteristic frequency (THz)	Vibrational mode
KcsA [15]	51.87	C=O bond stretching
Ca_V_Ab [16]	42.55	C–O bond stretching
K_V_1.2 [17]	1.4, 2.2 and 2.9	K^+^ vibration in *z*‐direction
	5.0	K^+^ vibration in *x*‐/*y*‐direction
	10.8	C=O wiggling in *z*‐direction
	51	C=O bond stretching
Na_V_1.5 [17]	2.5 and 10	Na^+^ vibration
	42.5 and 48.6	C–O bond stretching
Ca_V_1.3	2, 5, 8, and 16	Ca^2+^ vibration
	42.6 and 48.5	C–O bond stretching

Abbreviation: SF, selectivity filter.

Despite the insights gained, computer simulations have limitations. For example, the electric field strengths used in simulations are considerably higher than those in experimental conditions, and results may vary depending on the force field employed. Nonetheless, the calculated frequencies offer valuable references for future experimental studies and could contribute to advancing THz biology.

## AUTHOR CONTRIBUTIONS

**Lingfeng Xue**: Methodology (equal); formal analysis (lead); writing—original draft (lead); writing—review and editing (equal). **Zigang Song**: Methodology (equal); software (lead); writing—review and editing (equal). **Qi Ouyang**: Conceptualization (supporting); writing—review and editing (equal). **Chen Song**: Conceptualization (lead); supervision (lead); writing—review and editing (lead).

## CONFLICT OF INTEREST STATEMENT

The authors Lingfeng Xue, Zigang Song, Qi Ouyang, and Chen Song declare no conflict of interests.

## ETHICS STATEMENT

This article does not contain any studies with human or animal subjects performed by any of the authors.

## Data Availability

The MD plug‐in is available on the GitHub repository (ComputBiophys/GMX_ElectricField_Plugin). The simulation data are available upon reasonable request.
